# Long-term quality of life, psychological distress, and caregiver burden in octogenarians with LVAD: A single-centre experience

**DOI:** 10.1177/03913988241239236

**Published:** 2024-03-23

**Authors:** Jacopo D’Andria Ursoleo, Marina Pieri, Francesco Calvo, Savino Altizio, Mario Gramegna, Domenico Pontillo, Silvia Ajello, Anna Mara Scandroglio

**Affiliations:** 1 Department of Anesthesia and Intensive Care, IRCCS San Raffaele Scientific Institute, Milan, Lombardia, Italy; 2 Vita-Salute San Raffaele University, Milan, Lombardia, Italy; 3 Department of Acute Cardiac Care, IRCCS San Raffaele Scientific Institute, Milan, Lombardia, Italy

**Keywords:** Caregivers, left ventricular assist device, octogenarians, Psychological General Well-Being Index, quality of life, Short-Form-36 questionnaire

## Abstract

With the general population aging, both life expectancy and the number of left ventricular assist device (LVAD) implantations in elderly patients are growing. Nevertheless, their perceived long-term quality of life, including psychological aspects, coupled with the respective caregiver’s burden, remain under-reported. In light of the rising number of octogenarians with LVAD who necessitate broader healthcare provider involvement, we assessed the long-term quality of life, as defined by both the 36-item short-form health (SF-36) survey and the EuroQol 5 dimensions, 5-level questionnaire (EQ-5D-5L)—including the visual analog scale—in octogenarian LVAD patients who had received treatment at our institution. Additionally, we evaluated the psychological health of octogenarian LVAD patients using the psychological general well-being index (PGWBI), alongside their caregivers’ well-being through the 22-item version of the Zarit Burden Interview (ZBI). Of 12 octogenarian LVAD patients, 5 were alive and willing to answer questionnaires. Mean age at implant was 74 ± 2 years. Median follow-up was 2464 (IQR = 2375–2745) days. Although variable, the degree of health and psychological well-being perceived by octogenarian patients with LVAD was “good.” Interestingly, the burden of assistance reported by caregivers, though relevant, was greatly varied, suggesting the need to better define and address psychological long-term aspects related to LVAD implantation for both patients and caregivers with a broad-spectrum approach.

## Background

Left ventricular assist device (LVAD) implantation has become an established therapeutic option for advanced heart failure (AHF) patients who are not heart transplantation (HT) candidates. With the general population aging, both life expectancy and the number of LVAD implantations in patients aged ⩾70 years are growing.^1–3^ Namely, 1182 patients from the interagency registry for mechanically assisted circulatory support (INTERMACS) were recently identified as having >75 years at time of LVAD implant.^4^ Consequently, the number of octogenarians with LVAD as destination therapy (DT) is expected to increase.^1^ Surprisingly, while several reports regularly outline clinical outcomes of patients with continuous-flow durable LVAD, their perceived long-term quality of life (QOL), psychological and emotional implications, coupled with the res-pective caregiver’s burden, are under-reported and remain mostly unknown. As older adults receiving an LVAD are commonly frail patients displaying concomitant multiple chronic conditions, a call to gain insight on their perceived QOL and the emotional challenges arising from their condition, which would ultimately affect the success of LVAD therapy, is needed.^5^ We hereby describe the results of our observational follow-up study of octogenarian LVAD patients treated and followed up at our institution. We evaluated long-term quality of life in terms of both health status and psychological health as perceived by the patients, and conducted a burden interview with their caregivers.

## Methods

All patients alive and aged ⩾80 years at the time of this analysis who were implanted with an LVAD at the IRCCS San Raffaele Scientific Institute from 2010 (first device implanted) to August 2023, were included in the study. Data collection was performed with the approval of the Institutional Ethical Committee and with patients’ infor-med consent. Inclusion criteria also included having been implanted with an LVAD for at least 1 year and absence of cognitive or sensorial disorders preventing them from taking part in the assessment. Exclusion criteria were: patients who had undergone implantation of an LVAD for ⩾1 year but were aged <80 years at the time of assessment, LVAD implantation within the preceding year, patients explicitly refusing study participation, presence of concomitant cognitive or sensorial disorders impeding the conduct of patient assessments. All patients were regularly scheduled for follow up visits at our outpatients’ department as per institutional practice. In addition, a 24/7 phone line dedicated to LVAD patients was available to address any adjunctive clinical need.

QOL was assessed by the short-form 36 (SF36) of the medical outcomes study and the EuroQol 5 dimensions, 5-level questionnaire (EQ-5D-5L) including the visual analog scale (VAS). Both instruments were previously validated and used in studies on LVAD patients.^6^ The psychological general well-being index (PGWBI) scale was employed to assess psychological attributes associated with disease experience. Caregiving burden was assessed through the 22-item version of the Zarit Burden Interview (ZBI). The Cronbach’s alpha was good or excellent for every test. Clinical data were retrieved from records charts and stored electronically. Patients and caregivers answered personally to questionnaires. Data are presented as mean ± standard deviation, median, and interquartile range or frequency (%) as appropriate.

## Results

### Sample characteristics

Twelve patients (7%) of total LVAD implantations during the study period met the inclusion criteria and were included in the study. Table 1 summarizes the clinical characteristics of study patients. The indication for LVAD was destination therapy in all patients (100%). All patients who received durable LVAD between 2012 and 2018 had major comorbidities, as shown in Table 1. Mean age at implant was 74 ± 2 years, and all patients were male except for 1 (92%).

**Table 1.** Patients’ characteristics.

Patient	Gender	Comorbidities	Age at implant	Year of implant	INTERMACS class	Type of LVAD	Follow-up duration (d)	NYHA class at last available follow-up	Cause of death	Hospital re-admissions for complications	Number of hospital re-admissions	Causes for hospitalization
1	Male	Peripheral artery disease	73	2012	4	HM2	3060	2B	Acute renal failure	Yes	3	Right heart failure, infection, arrhythmia
2	Male	Chronic kidney disease and abdominal aortic aneurysm (surgery)	73	2013	4	HVAD	2433	2	Not available	Yes	3	Pump thrombosis, infection, arrhythmia
3	Male	Peripheral artery disease, chronic obstructive pulmonary disease, hypothyroidism, and ischemic stroke	71	2014	2	HVAD	3180	2B	Alive	Yes	11	Infection (site fistula), recurrent bleeding, heart failure, arrhythmia
4	Female	Hypothyroidism	77	2015	3	HVAD	2918	3B	Cardiovascular	Yes	7	Ischemic stroke, infection, recurrent bleeding, heart failure, arrhythmia
5	Male	Chronic kidney disease, gout, psoriasis, and gastroesophageal reflux disease	74	2016	3	HVAD	2687	2B	Alive	Yes	3	Right heart failure, infection, arrhythmia
6	Male	Chronic kidney disease and hypothyroidism	73	2016	2	HM3	2552	2B	Not available	Yes	11	Recurrent bleeding, arrhythmia
7	Male	Chronic hepatitis C infection, chronic atrial fibrillation, and chronic obstructive pulmonary disease	75	2016	3	HM3	2474	2B	Alive	No	0	None
8	Male	High blood pressure, dyslipidemia, type 2 diabetes mellitus, chronic kidney disease, and benign prostatic hyperplasia	73	2016	2	HM3	2453	3	Alive	Yes	2	Infection, arrhythmia
9	Male	High blood pressure and dyslipidemia	73	2017	4	HVAD	2115	2B	Not available	Yes	5	Infection, arrhythmia
10	Male	Chronic atrial fibrillation	75	2017	4	HVAD	2380	2	Alive	Yes	6	Recurrent bleeding, arrhythmia
11	Male	Cancer (prostate)	75	2017	1	HM3	2361	2A	Alive	Yes	6	Infection, recurrent bleeding
12	Male	Myelodysplastic syndrome, obstructive sleep apnea, chronic kidney disease, gastric ulcer, gout, deep vein thrombosis, pneumonia, and cancer (prostate)	76	2018	4	HM3	1749	2B	Multiorgan failure	Yes	8	Infection, arrhythmia, recurrent bleeding, heart failure

INTERMACS: interagency registry for mechanically assisted circulatory support; LVAD: left ventricular assist device; HM: heartmate; HVAD: HeartWare Ventricular Assist Device; d: days; NYHA: New York Heart Association.

Six patients had received HeartWare HVAD (Medtronic Inc., Mounds View, MN), five patients were implanted with HeartMate3 device (Abbott Labs, Chicago, IL), and one with HeartMate2 (Thoratec Corporation, Pleasanton, CA, USA).

Median duration of mechanical support to last available follow-up was 2464 (IQR = 2375–2745) days. Maximum recorded support duration was 3180 days, approaching 9 years of durable LVAD support in one patient. All patients experienced major complications over the prolonged period of LVAD support, requiring several hospital admissions (Table 1). Six patients died after 2493 (IQR = 2274–2827) days. The remaining six patients were asked to participate in the interview: most of them (*n* = 5) agreed to participate in the study and only one refused, due to severe health issues (hospitalization because of a hemorrhagic complication). All patients were native speakers of Italian and were interviewed in Italian.

### Quality of life, psychological and emotional well-being, and caregiver burden

Table 2 summarizes the scores of the questionnaires. Interestingly, we found a certain degree of variability in the reported physical and psychological health status as perceived by patients. While health perception ranged from 30% to 75% (with 100% being the best possible score), psychological and emotional well-being was higher overall: three out of five patients had >96/110 score and the other two patients scored 79/110 and 60/110, respectively. However, the highest heterogeneity was observed in the score assessing the caregiver burden (Table 2): two caregivers reported a minimum burden (ZBI < 20), one a mild-to-moderate burden (ZBI = 21–40), one a moderate-to-severe burden (ZBI = 41–60), and one a severe burden (ZBI 61–80). All the interviewed patients reported a feeling of trust with regard to the medical LVAD team. Three out of five patients indeed commented that, due to the dedicated LVAD phone line, they had the certainty that any relevant issue could be promptly managed by experienced clinicians and this greatly helped control their anxiety and feelings of depression.

**Table 2.** Questionnaires results.

	Patient 5	Patient 7	Patient 8	Patient 10	Patient 11
Short-Form-36 (SF-36)					
Physical function (PF)	35%	45%	60%	60%	80%
Social function (SF)	67%	44%	100%	100%	89%
Mental health (MH)	96%	64%	84%	96%	92%
Pain (P)	89%	67%	100%	100%	100%
Change in health (CiH)	50%	50%	100%	25%	50%
Role limitation—physical (RLP)	75%	0%	0%	0%	75%
Role limitation—mental (RLM)	100%	100%	100%	100%	67%
Energy/Vitality (EV)	60%	65%	70%	75%	80%
Health perceptions (HP)	50%	30%	60%	65%	75%
Psychological General Well-Being Index (PGWBI)					
Anxiety	21/25	17/25	25/25	25/25	24/25
Depressed mood	13/15	9/15	14/15	15/15	15/15
Positive well-being	9/20	7/20	15/20	16/20	19/20
Self-control	11/15	9/15	15/15	15/15	14/15
General health	12/15	7/15	10/15	12/15	15/15
Vitality	13/20	11/20	17/20	16/20	18/20
Total score	79/110	60/110	96/110	99/110	105/110
EQ-5D-5L					
Mobility	1	3	3	3	1
Self-care	3	2	2	2	1
Usual activities	2	3	2	3	1
Pain/discomfort	2	1	1	1	1
Anxiety/depression	1	3	1	1	1
EQ-VAS	75/100	50/100	50/100	80/100	80/100
QoL Caregivers—Zarit Burden Interview (ZBI)					
Total score	4/88	48/88	27/88	7/88	44/88

**Figure 1.**
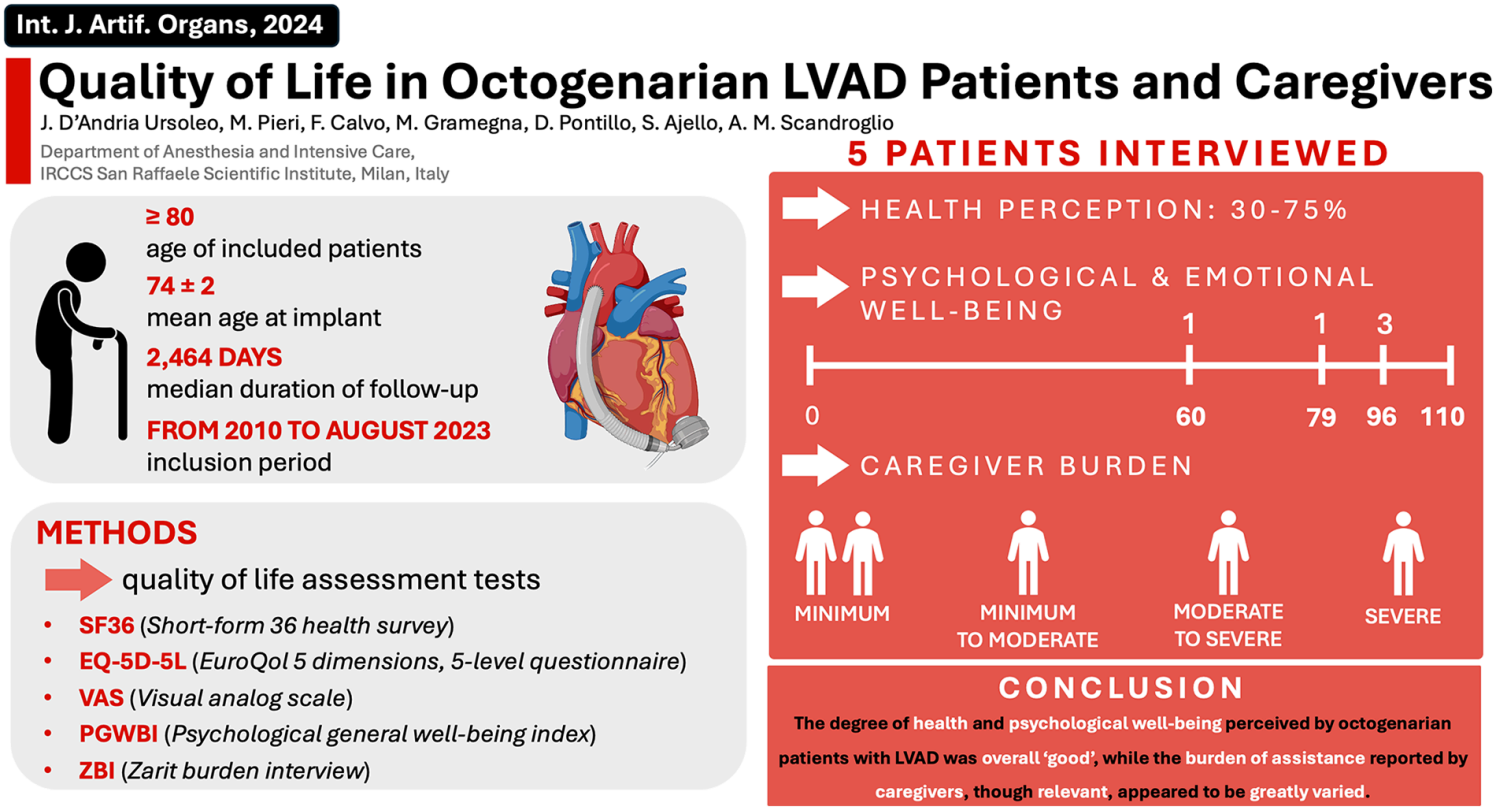
Visual abstract presenting main article background, objective, research methodology, and findings.

Figure 1 serves as a comprehensive visual representation of the core findings of our original investigation.

## Discussion

This is a pioneering preliminary study designed to investigate the long-term living experience of octogenarian LVAD patients. LVAD therapy can in fact meaningfully prolong life expectancy for LVAD patients aged ⩾80 years. Remarkably, if carefully selected for LVAD therapy, no specific clinical concerns can be expected *a priori* for octogenarians, compared to existing published data referring to LVAD patients aged ⩾65 years.^7^ However, beyond the strictly clinical issues, how does LVAD therapy affect the QOL of the very elderly population? To date, this aspect remains largely unknown. Indeed, if receiving an LVAD allows for enhanced physical performance status (e.g. expected survival) for elderly patients with acute heart failure, more research should focus on the psychological and emotional aspects of these patients and the burden on their caregivers, as this type of distress may potentially be sustained even in the long-term. Rossi Ferrario et al.^2^ recently documented the impact of post-discharge psychological difficulties on LVAD patients and their caregivers. Their observations were in line with current emerging literature emphasizing the necessity of addressing pertinent non-strictly-medical factors as part of the holistic care of long-term mechanical circulatory support (MCS) patients.^7^ Nonetheless, when it comes to octogenarians, age-specific outcome data essential for informing providers, patients, and caregivers in adults aged ⩾75 years are limited.^5^

Our investigation, although carried out on a very small group of patients, suggests that great heterogeneity exists in long-term perceived health status and quality of life of patients. Similarly, the caregiving load, as perceived by caregivers, is distributed from one extreme to the other. This topic is multifaceted and, until now, under-investigated. The cultural environment of patients may also vary greatly, along with the background of the caregiver (e.g. age, sex, level of instruction, socio-economic status, and degree of kinship with patients)—all these elements act concomitantly and play a relevant impact on patients’ long-term progress.

Addressing the quality of life of patients and limiting the caregiver burden is even more relevant in the very elderly, who are frail, more prone to complications and generally more dependent on third-party assistance.^8^ Awareness of such distinctive traits is of paramount importance for healthcare providers involved in elderly LVAD patient care, as it is anticipated that an increasing number of geriatric patients will be considered for LVAD support in the near future.^9^

Moreover, the ongoing evolution of technology presents an escalating set of challenges specifically for elderly patients. In this context, as technological advancements continue to emerge, the adoption of telemedicine and telemonitoring is growing, encompassing a wide array of medical devices, notably including MCS systems. However, digital and computer-based systems often pose difficulties for elderly patients due to their lack of skills in this area. This challenge is compounded by the fact that a growing proportion of caregivers are themselves part of the middle or senior age demographic. In order to comprehensively assess the ramifications of this increasingly digitized aftercare, it is imperative that more extensive and long-term studies be conducted involving larger patient cohorts to elucidate the potential impact of these evolving technological interventions on crucial aspects such as mortality rates, morbidity outcomes, overall QOL, and healthcare costs, to ultimately understand the holistic effects on elderly patients, caregivers, and the broader healthcare landscape.^10^

## Study limitations and future perspectives

Several limitations of our study warrant consideration. Firstly, we investigated a restricted sample size within a single setting and a one-time point measurement only. Nonetheless, our QOL findings align with those from multicenter reports, consistent with existing data pertaining to LVAD patients aged ⩾65 years. Secondly, while formal cognitive screening tests were not administered, we conducted clinical phone interviews as a viable means of remote evaluation. Thirdly, our study also features an underrepresentation of females. Furthermore, the small patient number likely contributed to the heterogenic results in the assessment of the QOL of patients as well as in the evaluation of the caregiver’s burden. Consequently, all of our findings should be considered as hypothesis-generating only.

## Conclusions

With the aging demographic and a growing LVAD patient population, understanding the challenges and nuances of their long-term care, especially in geriatric and octogenarian adults, becomes crucial. This encompasses not only medical considerations but also the psychological and emotional well-being of both patients and caregivers. Understanding and addressing the needs of aged patients and their caregivers that are not-strictly clinical may potentially represent a further step towards a more comprehensive multimodal, holistic, and patient-centered approach to LVAD care and support, enabling patients to age in dignity and comfort.

## References

[1] Yuzefpolskaya M Schroeder SE Houston BA , et al. The society of thoracic surgeons intermacs 2022 annual report: focus on the 2018 heart transplant allocation system. Ann Thorac Surg 2023; 115(2): 311–327. 36462544 10.1016/j.athoracsur.2022.11.023

[bibr2-03913988241239236] Rossi Ferrario S Panzeri A Pistono M . Psychological difficulties of LVAD patients and caregivers: a follow up over one year from discharge. Artif Organs 2022; 46(3): 479–490. 34519060 10.1111/aor.14071 PMC9292387

[3] Atluri P Goldstone AB Kobrin DM , et al. Ventricular assist device implant in the elderly is associated with increased, but respectable risk: a multi-institutional study. Ann Thorac Surg 2013; 96(1): 141–147. 23731606 10.1016/j.athoracsur.2013.04.010 PMC4111243

[4] Emerson D Chikwe J Catarino P , et al. Contemporary left ventricular assist device outcomes in an aging population: an STS INTERMACS analysis. J Am Coll Cardiol 2021; 78(9): 883–894. 34446160 10.1016/j.jacc.2021.06.035

[5] DeFilippis EM Nakagawa S Maurer MS , et al. Left ventricular assist device therapy in older adults: addressing common clinical questions. J Am Geriatr Soc 2019; 67(11): 2410–2419. 31400228 10.1111/jgs.16105

[6] MacIver J Ross HJ. Quality of life and left ventricular assist device support. Circulation 2012; 126: 866–874. 22891167 10.1161/CIRCULATIONAHA.111.040279

[7] Streur MM Auld JP Liberato ACS , et al. Left ventricular assist device caregiver experiences and health outcomes: a systematic review of qualitative and quantitative studies. J Card Fail 2020; 26(8): 713–726. 32505816 10.1016/j.cardfail.2020.05.018 PMC7484208

[8] Gazda AJ Kwak MJ Akkanti B , et al. Complications of LVAD utilization in older adults. Heart Lung 2021; 50(1): 75–79. 32709497 10.1016/j.hrtlng.2020.07.009

[9] Goldstein NE May CW Meier DE. Comprehensive care for mechanical circulatory support: a new frontier for synergy with palliative care. Circ Heart Fail 2011; 4(4): 519–527. 21772016 10.1161/CIRCHEARTFAILURE.110.957241 PMC3158989

[10] Schmidt T Mewes P Hoffmann JD , et al. Improved aftercare in LVAD patients: development and feasibility of a smartphone application as a first step for telemonitoring. Artif Organs 2020; 44(3): 248–256. 31435951 10.1111/aor.13560

